# Examination of the Effectiveness of a Universal Prevention Program to Enhance Understanding and Regulating Others’ Emotions for Children in Terms of Implicit and Explicit Affect

**DOI:** 10.1192/j.eurpsy.2023.676

**Published:** 2023-07-19

**Authors:** K. Uchida, K. Yamasaki

**Affiliations:** Department of Psychology and Educational Science, Naruto University of Education, Naruto, Tokushima, Japan

## Abstract

**Introduction:**

Uchida & Yamasaki (2012, 2022) have developed a universal prevention program to enhance understanding and regulating others’ emotions for elementary and junior high school students. In recent years, affect and emotions are popular research topics in the domains of psychology and brain science. Most research has thus far focused on the effects of explicit affect on health and adjustment. However, an increasing number of studies have started to examine the effects of implicit affect on psychological outcomes. Although the program was developed for enhancing coping of explicit emotions for health and adjustment, the effectiveness needs to be examined also in terms of implicit affect.

**Objectives:**

The purpose of this study was to examine the effectiveness of this program in terms of implicit and explicit affect.

**Methods:**

Participants were 6th-grade children in a public elementary school in Japan. The final sample was 61 children (32 boys and 29 girls). Participants completed a battery of two questionnaires just before (Time 1) and just after (Time 2) the intervention program. The questionnaires were the Implicit Positive and Negative Affect Test for Children (IPANAT-C) for assessing implicit positive and negative affect (IPA and INA) and the Japanese version of the Positive and Negative Affect Schedule for Children (PANAS-C) for measuring explicit positive and negative affect (EPA and ENA). The universal intervention program that was one of the programs we developed for children’s health and adjustment was implemented over four regular classes targeting all children in their homeroom classes.

**Results:**

Data were analyzed by 2 (pre-intervention and post-intervention periods) x 2 (boys and girls) analyses of variance (ANOVA) with the hoc tests using Holm corrections. First, regarding the EPA and IPA, there was a significant main effect of periods (*F*s (1, 59) = 6.82 and 4.54, *p* < .05, respectively), revealing in the post hoc tests that they significantly increased at the post-intervention period. In contrast, regarding ENA and INA, there was no significant main effect of periods. Moreover, regarding EPA, there was a significant main effect of sex. No significant period x sex interactions were found in any affect.

**Conclusions:**

These results revealed that the current program was effective in enhancing explicit and implicit positive affect. On the other hand, no significant effects were found in enhancing explicit and implicit negative affect. The necessity of future research that will examine the sustainability of the effectiveness of the program is discussed, along with several limitations.

**Disclosure of Interest:**

None Declared

